# Chemobrain, Olfactory and Lifestyle Assessment in Onco-Geriatrics: Sex-Mediated Differences between Chemotherapy and Immunotherapy

**DOI:** 10.3390/brainsci12101390

**Published:** 2022-10-14

**Authors:** Sara Invitto, Mariangela Leucci, Giuseppe Accogli, Andrea Schito, Claudia Nestola, Vincenzo Ciccarese, Ross Rinaldi, Paolo Boscolo Rizzo, Giacomo Spinato, Silvana Leo

**Affiliations:** 1 INSPIRE LAB-Laboratory of Cognitive and Psychophysiological Olfactory Processes, DiSTeBA, University of Salento, 73100 Lecce, Italy; 2 Department of Medical Oncology, Vito Fazzi Hospital, 73100 Lecce, Italy; 3 Istituto Santa Chiara, Via Campania 5, 73100 Lecce, Italy; 4 Department of Mathematics and Physics “Ennio De Giorgi”, University of Salento, Via Monteroni, 73100 Lecce, Italy; 5 Department of Medical, Surgical and Health Sciences, Section of Otolaryngology, University of Trieste, 34129 Trieste, Italy; 6 Section of Otolaryngology, Regional Centre for Head and Neck Cancer, Department of Neurosciences, University of Padova, 31100 Treviso, Italy

**Keywords:** chemotherapy, immunotherapy, chemobrain, cognitive impairment, olfaction, frailty

## Abstract

A possible link between chemotherapy and cognitive impairment has been identified. In the literature, this condition is usually called chemobrain and can mostly affect some memory domain but can lead also to other cognitive impairments. Olfaction, which is known to be linked with cognitive domain and the nociception system, can also be affected by chemotherapy. The aim of this study was to investigate the main cognitive and olfactory abilities and the functional and nutritional state of a cohort of chemotherapy and immunotherapy onco-geriatric patients and control geriatrics subjects. Cognitive, olfactory, geriatric and nutritional assessments were performed through the Mini Mental State Examination (MMSE), Sniffin’ Sticks Screening 12, G8 test and a questionnaire on the adherence of the Mediterranean diet, respectively. Our findings show a gender effect on the MMSE. Overall results indicate more pronounced impairments both at the cognitive and frailty level regardless of the type of therapy. On the other hand, the Sniffin’ Sticks performances highlight a significant decrease in olfactory perception ability of subjects following immunotherapy. Significant correlations between olfactory performance and MMSE and G8 scores were also found, as well as between MMSE and G8 measures.

## 1. Introduction

### 1.1. The Chemobrain and Oncologic Therapy

Recently, there is an increasing number of studies that show a certain level of decline in the cognitive functions of people being treated for cancer, although some of these cases do not completely fulfil the criteria for mild cognitive impairment [1]. Evidence suggests that these deficits mostly affect chemotherapy subjects [2,3]. Depending on the studies, this type of cognitive impairment occurs with an incidence rate ranging from 16% to 75% of cases [4,5].

These effects on the cognitive system are defined in the scientific literature as chemotherapy-induced cognitive impairment (CICI), chemotherapy-related cognitive impairment (CRCI), chemofog or chemobrain and tend to reflect on memory, learning, executive functions, attention and visuo-spatial skills [6,7]. In particular, Downie and colleagues [8] reported short-term memory criticalities, an increase in information retrieval time and difficulties related to the ability to concentrate, to verbal fluency, to words research, to processing speed and, to a lesser extent, to planning and visual-spatial skills.

Moreover, further investigations [9,10] have also highlighted the effects on working memory and critical issues affecting multitasking skills. The impairment could also involve prospective memory [11] due to damage to the connections between the prefrontal cortex and white matter caused by adjuvant chemotherapy [12]. Regarding working memory (WM), Ferguson and colleagues [13] highlighted a greater activation of the circuit structures dedicated to WM in subjects who had undergone chemotherapy.

A significant impairment of visual memory was also highlighted [14].

Chen and colleagues [15] demonstrated how chemotherapy subjects show great difficulty maintaining a state of alert for a long period of time and in resolving and responding to conflicts between competing information.

However, elderly subjects with a low level of cognitive reserve before chemotherapy treatment seem to be more sensitive to the effects of chemotherapy on the cognitive system [16,17].

The duration of the chemobrain is variable. Some analyses report a symptomatology of a few months that can last also for ten years or more after treatment [3]. Nevertheless, some studies underline that it seems to be more acute during the course of chemotherapy treatment [4] with the chance of a subsequent weakening once it has ceased [6].

In addition, the strong distress related to the cognitive symptoms experienced by cancer patients who report that they cannot adequately carry out daily or work activities as they could before the disease or that they can only perform them through a more intense mental effort should not be underestimated [18].

There may be many causes that lead to chemobrain, and although they are still unclear, the presence of multiple factors capable of contributing to the phenomenon described above have been hypothesized as possible causes [19].

First of all, it is necessary to underline that the presence of a cognitive deficit could be detected not necessarily during or after chemotherapy treatment, but also after the diagnosis of cancer but before starting therapy [20]. Therefore, it cannot be excluded that cancer itself, in some way, may be a sufficient factor for the appearance of cognitive symptoms. Psychological reactions to cancer diagnosis can also influence cognitive performance. Anxiety and depression can occur most often with the diagnosis of cancer [21,22] and can be causes or contributing factors to chemobrain. In this regard, Hurria and colleagues [23] highlight the presence of significant distress in 41% of geriatric cancer patients, suggesting that anxiety and depression can be risk factors for cognitive impairment in cancer patients [1]. Moreover, it has been shown that cancer-related fatigue, which can persist for over five years after the end of treatment, can lead to a decrease in attention, concentration, motivation and energy and can compromise an individual’s functional skills [24].

From a neurobiological point of view, cancer and chemotherapy could alter white and grey matter, thus affecting the brain’s structure and function [25,26] as well as reducing the frontal and temporal cortex [26]. Moreover, chemotherapy agents could have harmful effects on mature neural cells and vascular structures [27].

Among the possible causes and mechanisms that induce chemofog, the role of hormonal changes is still to be considered. In fact, a reduction in cognitive functions has been found in women treated not only with hormonal therapy but also in combination with chemotherapy [28,29].

Furthermore, most of the agents used for chemotherapy do not generally cross the blood–brain barrier, but some animal studies have shown that very low doses of chemotherapy agents can cause cell death and a reduction in cell division within brain structures important in cognition [30].

In parallel with chemofog studies, research on the effects on the cognitive system of other types of cancer treatment are also starting to increase. In particular, research has shown that some immunotherapeutic agents, especially when combined together, could increase the risk of severe toxicity [31] and if combined with chemotherapy or biological therapy, compared with only chemotherapy treatment, are associated with increased treatment-related mortality [32].

Furthermore, immunotherapy seems to be connected to neuropsychological changes in cancer patients. Immunotherapy, especially in combination with other treatments (such as peripheral radiotherapy), may lead to an increase in the presence of neurological dysfunctions [33,34,35]. Moreover, immunotherapy seems to be involved in a number of pathologies that involve all areas of the central and peripheral nervous systems [36]. In particular, immunotherapy uses the patient’s immune system to fight cancer instead of directly targeting the tumor. Immunotherapy treatments include cancer vaccines, oncolytic viruses, adoptive transfer of ex vivo activated T and natural killer cells and the administration of antibodies or recombinant proteins that either stimulate the cells or block the immune checkpoint pathways [37]. Hence, immunotherapy seems to increase the anti-tumor immune responses through the expansion of T cells reactive to the tumor, providing exogenous stimuli of immune activation and antagonistic regulatory pathways [38]. For example, the IFN-alpha cytokine is used to induce a natural immunologic response against malignant tumors, but impairments of verbal memory, executive functioning and psychomotor speed can be found after treatment, especially if it used in combination with chemotherapy [39,40].

However, neurological disorders, including cognitive impairment, appear to be rare in immunotherapy but could occur more severely and, nevertheless, there is a small number of studies about them [35].

### 1.2. Chemosensory Functions and Chemobrain

Olfaction is essential for chemosensory perception and allows for the direction of attention towards environmental risks or odors that usually have positive connotations, such as food [41]. Therefore, the olfactory system is capable of influencing behavior, awareness of environmental risks and even social communication [42].

Olfactory disorders can be categorized through perceptual symptoms; therefore, it is possible to distinguish among: dysosmia, i.e., the difficulty in identifying odors; parosmia, i.e., the sensation of a smell different from that typical of a certain substance; phantosmia, i.e., the inability to perceive odors; hyposmia, i.e., a reduced ability to perceive smell; hyperosmia, i.e., an exaggerated sensitivity of the sense of smell [43,44]. Chemosensory alterations are reported by about 86% of cancer patients [45]. In detail, chemotherapy generally affects rapidly dividing cells. Since the receptors responsible for the sense of smell (but also of taste) proliferate rapidly, they can be sensitive to the cytotoxicity of chemotherapy, too [46]. Tests on animal samples have shown how specific chemotherapy drugs can cause functional changes in the olfactory epithelium and, consequently, changes in electrophysiological responses, confirming changes in olfactory functions [47] and apoptosis in olfactory epithelium [48]. Olfactory changes can have consequences on an individual’s quality of life, affecting, for example, cooking, nutrition, safety and even personal hygiene [49,50], and they can even cause depressive manifestations, especially in the first months of olfactory impairment [51,52]. In this regard, Walliczek-Dworschak and colleagues [53] highlighted a significant correlation between depressive symptoms and the scores of the olfactory evaluations after the end of chemotherapy, confirming that the olfactively compromised patients showed peculiar signs of depression. Finally, it is assumed that a genetic predisposition may play a significant role in determining and perhaps even predicting the cognitive decline typical of chemobrain: cancer survivors with at least one e4 allele of apolipoprotein E (APOEe4) seem to be more likely to manifest significant cognitive deficits than APOEe4 non-carriers [7,54]. In this context, the sense of smell could play an important role in the identification of cancer patients who may develop cognitive deficits. In fact, it has already been shown how the impairments of olfactory skills are correlated with the deterioration of the main cognitive functions of an individual (memory in particular), as far as to be able to predict the development of mild cognitive impairment (MCI) in elderly subjects without manifestations of cognitive impairments [55,56,57] and to consider the olfactory assessment as an important tool and marker (among the others) for the conversion, in some cases, from MCI to clinical dementia [58,59]. In addition, the olfactory system also shows connections with the nociceptive system. In fact, certain odorants seem to activate both trigeminal and olfactory neurons [60,61]. Indeed, the orbitofrontal and rostral insular cortex seems to amplify the trigeminal input, and this amplification is absent in subjects with olfactory loss [61]. The loss of olfactory sensitivity could be associated with a reduced sensitivity to trigeminal stimuli, and the alteration of the intranasal trigeminal function seems to be stronger in subjects suffering from post-traumatic anosmia [62]. Some studies also suggest a link between nociception and smell at the ion channel level [63,64].

### 1.3. Geriatric and Nutritional Aspects of the Elderly Oncological Patient

In this context, it could be useful to identify the frail patients in order to implement a more appropriate therapeutic intervention plan [65], and this evaluation should be performed according to a multidimensional perspective and with a regular follow-up [66]. Cancer and its treatment also alter an individual’s metabolism. Furthermore, as previously pointed out, deficiencies in the chemosensory perception can alter the eating habits of patients, geriatric patients particularly. Nutritional status can be compromised in a large number of elderly cancer patients, and weight loss, above all, is an unfavorable factor of the likely course of the disease in patients undergoing chemotherapy [67]. A nutritional evaluation—and, consequently, an intervention—allows for the control of symptoms related to cancer, the reduction of post-operative complications and the rate of infections, improved treatment tolerance and the immune–metabolic response [68]. In this regard, it has been highlighted how adherence to the Mediterranean diet reduces the mortality rate linked to cardiovascular disorders and cancer [69]. For this reason, according to a holistic and multidisciplinary perspective, it is essential to use tools that can assess the adherence to the Mediterranean diet of the cancer patient. Starting from these premises, the aim of the present study is to examine the possible presence and the grade of cognitive and olfactory impairment in geriatric cancer patients, also with a gender perspective, treated with chemotherapy and immunotherapy, taking into account––according to a multidisciplinary perspective—functional, psychological and nutritional aspects.

## 2. Materials and Methods

### 2.1. Participants

The research involved both the Oncology Center G. Paolo II of P.O. Vito Fazzi of Lecce (Apulia, Italy) and the Laboratory of Cognitive and Psychophysiological Olfactory Processes of University of Salento. Onco-geriatric metastatic patients (mean age 78 ± 5.6) were recruited and subdivided as follows: 70 (Group 1; 30 women) treated with chemotherapy, 43 with immunotherapy (Group 2; 10 women) and 41 geriatric control subjects (Group 3; 22 women). All the patients followed palliative cares and suffered from neoplastic pathologies (i.e., gastrointestinal and lung cancer). The patients recruited underwent only chemotherapy or immunotherapy; they did not carry out any other type of treatment. They had not been the subject of either radiotherapy or surgery. They had not undergone neo-adjuvant treatments. The experimental study was approved by the Vito Fazzi AUSL LE Ethical Committee and by the IRB of DiSTeBA, University of Salento.

### 2.2. Assessment

The Mini Mental State Examination was used as a screening tool to evaluate the possible presence of cognitive impairment. The MMSE [70,71] is a 30-items test that allows for the assessment of some of the main cognitive areas, i.e., spatial and temporal orientation, memory skills (such as words recording and recall), attention, calculus, language and constructive praxis. Scores above the cut-off (22) indicate the absence of impairments. The test was used for research purposes and not to make diagnosis within specific nosological criteria.

The olfactory evaluation was carried out by The Sniffin’ Sticks Screening 12 (MediSense—http://www.medi-sense.eu) (accessed on 12 July 2021). The test consists of 12 odor pens and distinguishes anosmics and hyposmics from normosmics. The purpose of the test is to identify the aromas presented, and the patients are offered four options to choose from. The total score, which is higher for normal olfactory abilities, is compared to the age-related normative values.

The G8 test was used as a screening tool for geriatric assessment. This test includes seven items that measure appetite, weight loss, body mass index, motricity, self-related health, medication, cognition and depression [72]. The total score ranges from 0 to 17 (cut-off 14), where lower results represent greater frailty. The G8 is a simple and rapid instrument for identifying patients with a geriatric risk profile [73] and, even if it has a poor specificity, it presents one of the highest sensitivities for frailty [74].

Finally, a validated questionnaire on the evaluation of the adherence of the Mediterranean diet was administered in which lower values indicate higher non-adherence [75].

### 2.3. Statistical Analysis

JASP 0.16.1 software (University of Amsterdam, Amsterdam, The Netherlands) was used for statistical data analysis. A 2 × 4 multivariate ANOVA design was used in order to explore the influence of the type of therapy and gender (independent variables) on the scores obtained from the various tests (dependent variables). In addition, a correlation analysis was performed between all tests by Pearson’s coefficient. Statistical significance was set at *p* ≤ 0.05.

## 3. Results

**Figure 1:**
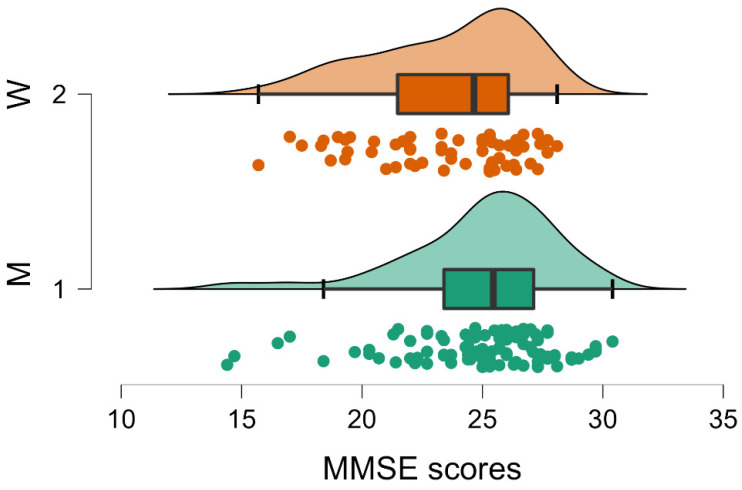
Raincloud plot of gender differences on MMSE showing significantly lower scores (*p* < 0.001) for women (W, Group 2) compared to men (M, Group 1). Median, interquartile range and maximum and minimum scores are represented by the thick vertical line, the box and the right and left whiskers, respectively. Curves and individual dots represent the data distribution.

**Figure 2:**
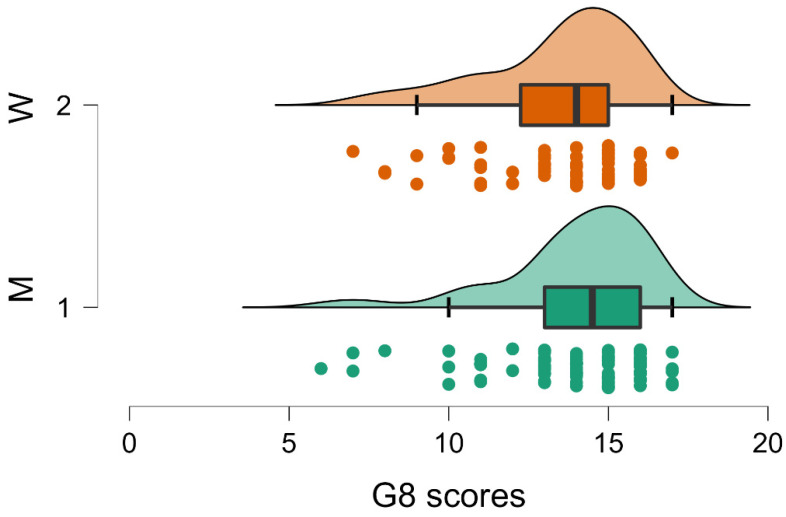
Raincloud plot relative to the trend toward significance (*p* = 0.054) of gender variable on G8 measures. Higher scores (i.e., lower frailty) were reported for men (M, Group 1). Conventions as in Figure 1.

MANOVA results showed significant differences related to gender (*p* < 0.001; Table 1). In particular, output from individual ANOVA per dependent variable (i.e., for each single test) resulting from the same analysis highlighted a significant effect for sex in MMSE (*p* < 0.001; F 13.618; Table 2, Figure 1), and a similar trend has been observed in G8 (*p* = 0.054; F 3.785; Table 2, Figure 2).

No significant difference related to gender was observed from the Sniffin’ Sticks Test or from the questionnaire on the evaluation of the adherence of the Mediterranean diet (Sniffin’ Sticks: *p* = 0.485; F 0.490; Mediterranean diet: *p* = 0.202; F 1.642; Table 2).

Regarding the significant gender effect on the MMSE and the trend on the G8, it was possible that men (Group 1) obtained higher scores both on the MMSE (men MMSE = 24.963; SD 3.154; women MMSE 23.587; SD 3.151; Table 3) and on G8 (men G8 = 14.011; SD 2.265; women G8 = 13.452; SD 2.317; Table 3). These results are in direction of a higher frailty for women.

**Figure 3:**
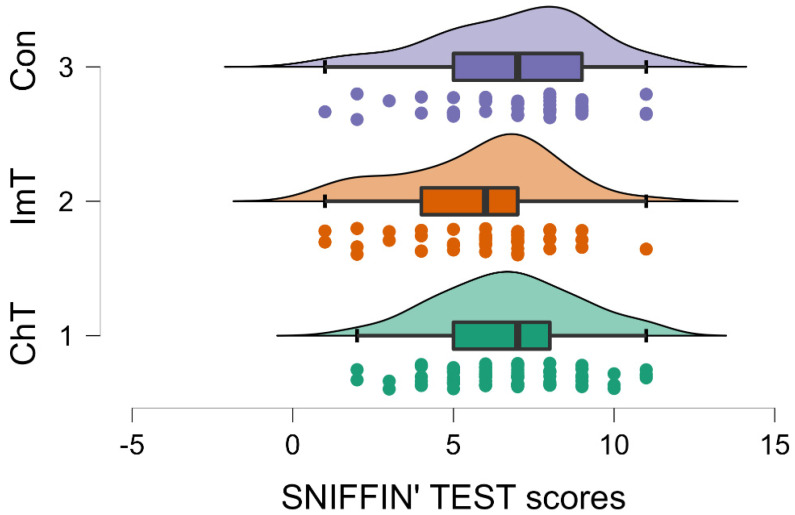
Raincloud plot showing the Sniffin’ Test scores of Group 1 (i.e., chemotherapy; ChT), Group 2 (i.e., immunotherapy; ImT) and Group 3 (i.e., geriatric control group). Patients treated with immunotherapy showed lower scores (*p* = 0.05) compared to chemotherapy and controls. Conventions as in Figure 1.

Moreover, the results highlighted a significant effect for therapy on the Sniffin’ Sticks Test (*p* = 0.050; F (1,2) = 3.054, Table 2). Patients treated with chemotherapy (Group 1) and control subjects (Group 3) showed higher scores than subjects treated with immunotherapy (Group 2) (Group 1: SNIFFING 6.729; SD 2.153; Group 2: SNIFFING 5.791; SD 2.356; Group 3: SNIFFING 6.854; SD 2.424; Table 2 and Table 4, Figure 3). This trend of data indicates a higher incidence of anosmia in immunotherapy patients. No differences related to the type of therapy emerged from MMSE, G8 or the questionnaire on the evaluation of the adherence of the Mediterranean diet. The descriptive analysis for each test in each group are reported in Table 4.

**Figure 4:**
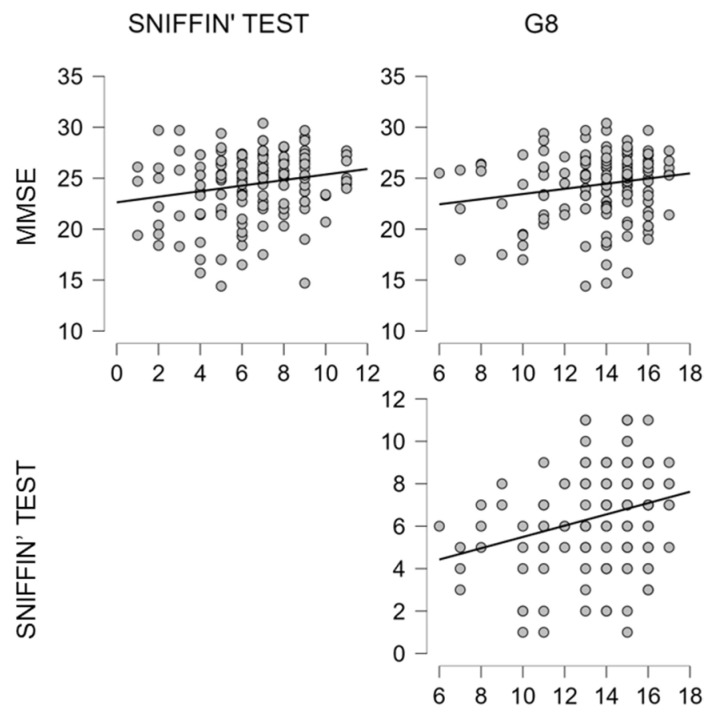
Scatterplots show significant correlations, as reported in Table 5, between MMSE, Sniffin’ test and G8.

Concerning the correlation analysis (see Table 5, Figure 4), a significant relationship was found between MMSE and olfactory performance (r = 0.226; *p* = 0.005), as well as between MMSE and the frailty measure on G8 (r = 0.163; *p* = 0.045). The higher correlation was found between the Sniffin’ Sticks and G8 (r = 0.279; *p* = <0.001).

## 4. Discussion

Frailty could include concepts such as the dependence and the risk of dependence of the elderly patient on others, the presence of complex medical, psychosocial conditions, chronic disease, important disabilities, weakness, weight loss and decreased physical activity [76,77]. Therefore, geriatric evaluation in the oncological field allows for the development of an integrated and coordinated individual treatment plan that can take into consideration the medical, psychosocial and functional aspects of the elderly person. Hence, the geriatric assessment examines a series of domains that have a particular impact on the quality of life of the elderly person, i.e., physical functionality, the presence of comorbidities, polypharmacy, nutrition, cognitive function and psychological status. So, this assessment is able to influence the decision-making process for cancer treatments and the management of these patients [78,79,80,81]. The evaluation of all of these aspects represents an opportunity to improve the support and the rehabilitation of the elderly. Furthermore, the literature reports how chemotherapy can have repercussions on specific cognitive abilities in patients treated with this therapy [8]. Furthermore, changes in olfactory perception are more frequent in older cancer patients [82], for whom a change in diet, a great reduction in appetite and food appreciation, poor nutritional status, changes in weight and greater risk of chronic diseases has been reported [49]. Olfactory abilities seem to decrease especially during chemotherapy treatment, and they appear to resolve a few months after the end of chemotherapy. Nevertheless, the impact and the influence of chemosensory changes on the patient’s nutritional status and quality of life should not be underestimated [82,83]. This framework seems to get worse when the olfactory deficits are connected to impaired taste perception [84]. Many describe a gradual process of deterioration and impoverishment of the chemosensory functions, although they are unable to specify the exact moment that it started [85]. The degree of the distress it causes, as well as the impact on daily life, tends to vary based on gender [86]. However, despite this, the present study shows that the scores obtained by the patients treated with chemotherapy in MMSE did not differ significantly from those of the control group and those who underwent immunotherapy. As for the latter, some immunotherapeutic agents can have negative consequences on the cognitive system causing fatigue and the manifestation of psychiatric symptoms [39,40]. Even in this case, immunotherapy does not appear to significantly affect the cognitive performance of patients compared to either the control group or the group of patients treated with chemotherapy. One possible explanation is provided by Hutchinson and colleagues [87]. Indeed, it has been found that, through subjective measurements of the deficit (self-reports, questionnaires, etc.), cancer patients often report memory or multitasking difficulties during daily activities.

## 5. Conclusions

Our study indicates that immunotherapy, assessed through the classical neuropsychological screening, does not affect cognitive domain, even if self-reported measures showed a stronger compromission. These perceptions could be related to quality of life, anxiety, depression and fatigue, since the assessments performed using neuropsychological batteries show a less prevalent cognitive deterioration than that detected by the self-reports. Olfactory perception, on the other hand, seems to be significantly influenced by the type of therapy to which one is subjected. In fact, patients treated with chemotherapy and the control group performed significantly better than those treated with immunotherapy. The literature shows that, in particular, chemotherapy, as a non-selective systemic treatment, in addition to acting on cancer cells, also acts on rapidly growing non-cancerous cells, such us mucous membranes and olfactory and gustatory receptors [83]. Indeed, the subjects in the control group scored higher in the olfactory identification test, but the significant difference was found when they were compared to the patients with immunotherapy.

A significant gender effect was found on the MMSE scores, and a trend was observed on the G8 scores. As for the former, men scored higher regardless of the group they belonged to. Indeed, the literature has shown that women’s mean MMSE scores decrease significantly faster with age than those of men [88]. The same trend was observed in the scores obtained by the women on the G8, in contrast with some studies that point out no gender risk factor for functional disability in the elderly [89]. Nevertheless, research by Sentandreu-Mañó and colleagues [90] highlights some manifestations of frailty that may differ by gender. For example, female sex was associated with lower physical and psychological quality of life. Furthermore, our results from correlation analysis, albeit of an exploratory nature, show how frailty measures are related to cognitive and olfactory abilities and that the latter two are correlated. This suggests that the olfactory assessment could be used as a very simple and sensitive tool for onco-geriatric patients manifesting cognitive complaints [56,57]. Such an approach might be useful in the future for validating patients’ experiences of changes and alterations due to cancer and its treatments, potentially avoiding in some cases the burdensome administration of neuropsychological tests which can also run into cases of underestimation. Limitations of the study include the small sample size and the use of a single test for evaluating cognitive functions. Furthermore, the COVID-19 pandemic did not allow us to recruit other subjects and continue research. This research reveals some future possibilities. Indeed, it might be worth analyzing how chemotherapy and immunotherapy affect olfactory perception with other neuroimaging instruments (e.g., EEG). Furthermore, in the future, the possible presence of cognitive deficits could be assessed with further sensorial, behavioral, cognitive and psychophysiological analyses in clinical aging. In this way it could be confirmed whether only gender or olfactory impairment can be considered as risk factors (i.e., gender and sex) or as a biomarker (i.e., olfactory impairment) in cognitive abilities in the geriatrics population undergoing anticancer treatments.

## Figures and Tables

**Table 1:** MANOVA results show a significant effect of gender (sex) on the dependent variables, i.e., the scores to the various tests.

Cases	df	Approx. F	Trace Pillai	Num df	Den df	*p*
THERAPY	2	1.108	0.061	8	280.000	0.358
SEX	1	5.172	0.130	4	139.000	<0.001
THERAPY ✻ SEX	2	0.560	0.032	8	280.000	0.810
Residuals	142					

**Table 2:** Individual ANOVA results per dependent variable (i.e., each single test) showing the significant effect of gender on MMSE performance, the trend on G8 and the significant effect of therapy on the Sniffin’ test.

	Cases	Sum of Squares	df	Mean Square	F	*p*
**MMSE**	THERAPY	22.509	2	11.255	1.257	0.288
	SEX	121.966	1	121.966	13.618	<0.001
	THERAPY ✻ SEX	2.181	2	1.090	0.122	0.885
	Residuals	1271.776	142	8.956		
**G8**	THERAPY	1.569	2	0.784	0.163	0.850
	SEX	18.195	1	18.195	3.785	0.054
	THERAPY ✻ SEX	4.849	2	2.425	0.504	0.605
	Residuals	682.685	142	4.808		
**Sniffin’ Sticks**	THERAPY	31.685	2	15.842	3.054	0.050
	SEX	2.541	1	2.541	0.490	0.485
	THERAPY ✻ SEX	16.242	2	8.121	1.566	0.213
	Residuals	736.525	142	5.187		
**Diet**	THERAPY	3.261	2	1.630	0.481	0.619
	SEX	5.570	1	5.570	1.642	0.202
	THERAPY ✻ SEX	1.132	2	0.566	0.167	0.847
	Residuals	481.787	142	3.393		

**Table 3:** Descriptive statistics for men (Group 1) and women (Group 2) for each test. Differences in scores on MMSE are significant.

	MMSE		SNIFFIN’ TEST		G8		DIET	
	1	2	1	2	1	2	1	2
Valid	92	62	92	62	92	62	86	62
Missing	0	0	0	0	0	0	6	0
Mean	24.963	23.587	6.315	6.774	14.011	13.452	7.884	7.565
Std. Deviation	3.154	3.151	2.223	2.432	2.265	2.317	1.704	1.989
Minimum	14.400	15.700	1.000	1.000	6.000	7.000	4.000	4.000
Maximum	30.400	28.100	11.000	11.000	17.000	17.000	13.000	15.000

*Note.* Excluded 4 rows from the analysis that correspond to the missing values of the split-by variable SEX.

**Table 4:** Descriptive statistics for MMSE, Sniffin’ Test, G8 and Diet scores for Group 1 (i.e., chemotherapy), Group 2 (i.e., immunotherapy) and Group 3 (i.e., geriatric control group).

	MMSE			SNIFFIN’ TEST			G8			DIET		
	1	2	3	1	2	3	1	2	3	1	2	3
Valid	70	43	41	70	43	41	70	43	41	67	40	41
Missing	0	0	0	0	0	0	0	0	0	3	3	0
Mean	24.276	23.942	25.127	6.729	5.791	6.854	13.843	13.767	13.707	7.746	7.550	7.951
Std. Deviation	3.253	3.375	2.918	2.153	2.356	2.424	2.124	2.213	2.686	1.726	1.632	2.167
Minimum	14.400	14.700	17.000	2.000	1.000	1.000	7.000	7.000	6.000	4.000	4.000	4.000
Maximum	29.700	30.400	29.700	11.000	11.000	11.000	17.000	16.000	17.000	13.000	10.000	15.000

**Table 5:** Pearson’s coefficient and and *p*-value for significant correlations are reported.

Variable		MMSE	SNIFFIN’ STICKS	G8
MMSE	Pearson’s r *p*-value	–		
SNIFFIN’ STICKS	Pearson’s r *p*-value	0.226 ** 0.005	–	
G8	Pearson’s r *p*-value	0.163 * 0.045	0.279 *** <0.001	–

* *p* < 0.05, ** *p* < 0.01, *** *p* < 0.001.

## Data Availability

Data are available upon request from the corresponding author who can be reached at sara.invitto@unisalento.it.
